# Supplementary material for: Diversity of post-translational modifications and cell signaling revealed by single cell and single organelle mass spectrometry
Source: Commun Biol. 2024 Jul 19;7:884. doi: 10.1038/s42003-024-06579-7 (PMC11271535; doi:10.1038/s42003-024-06579-7)
Supplement: Supplementary file 2 — Supplementray Information [file 42003_2024_6579_MOESM2_ESM.pdf]

Diversity of post-translational modifications and cell signaling revealed by single cell and single organelle mass spectrometry

Dong-Gi Mun[1], Firdous A. Bhat[1], Neha Joshi[1,2], Leticia Sandoval[1,3], Husheng Ding[1], Anu Jain[1], Jane A. Peterson[4], Taewook Kang[1], Ganesh P. Pujari[1], Jennifer L. Tomlinson[5], Rohit Budhraja[1], Roman M. Zenka[4], Nagarajan Kannan[1], Benjamin R. Kipp[1], Surendra Dasari[6], Alexandre Gaspar-Maia[1,3], Rory L. Smoot[5,6], Richard K. Kandasamy[1,7,§] and Akhilesh Pandey[1,2,3,§]

[1]Department of Laboratory Medicine and Pathology, Mayo Clinic, Rochester, MN 55905, USA
[2]Manipal Academy of Higher Education, Manipal, 576104, Karnataka, India
[3]Center for Individualized Medicine, Mayo Clinic, Rochester, MN 55905, USA
[4]Proteomics Core, Mayo Clinic, Rochester, MN 55905, USA
[5]Department of Surgery, Mayo Clinic, Rochester, MN 55905, USA
[6]Department of Biochemistry and Molecular Biology, Mayo Clinic, Rochester, MN, USA
[7]Department of Quantitative Health Sciences, Mayo Clinic, Rochester, MN 55905, USA

[§]Correspondence should be addressed:

Akhilesh Pandey, M.D., Ph.D.
Department of Laboratory Medicine and Pathology, Mayo Clinic
200 First St SW, Rochester, MN 55905
Email: pandey.akhilesh@mayo.edu

Richard K. Kandasamy, Ph.D.
Department of Laboratory Medicine and Pathology, Mayo Clinic
200 First St SW, Rochester, MN 55905
Email: kandasamy.richardkumaran@mayo.edu

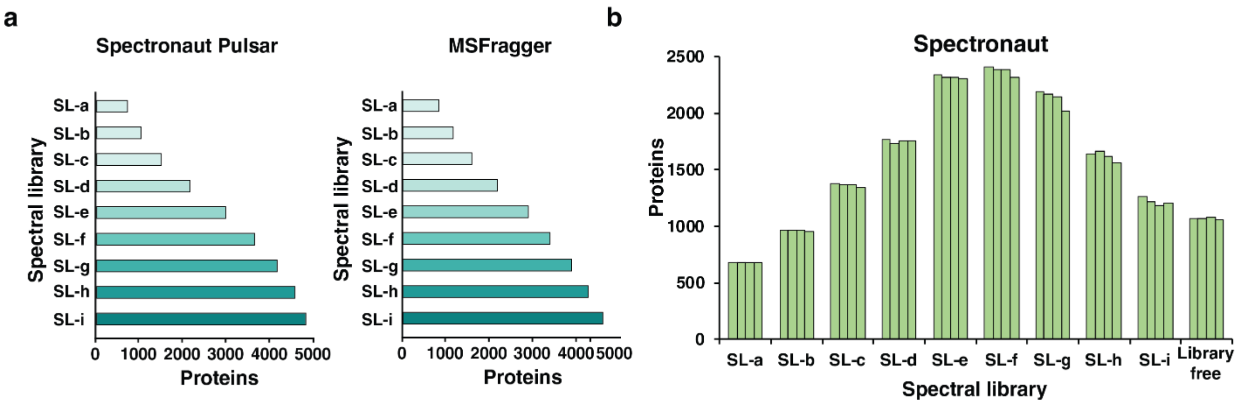

Figure S1. Optimum spectral library for analyzing diaPASEF data of single cells. **(a)** The number of proteins of spectral libraries generated using Spectronaut Pulsar or MSFragger using DDA-PASEF data described in figure1a. **(b)** The number of proteins identified from diaPASEF data of 156 pg peptides using Spectronaut.

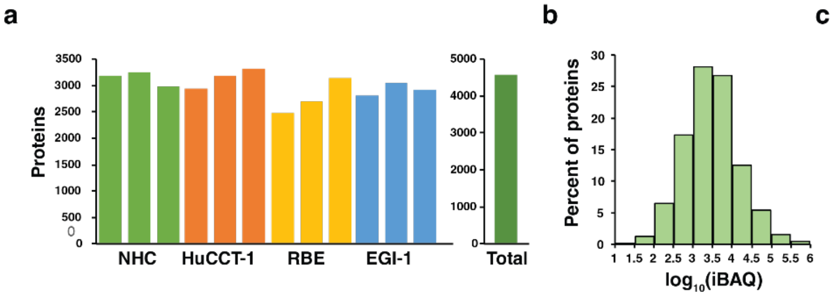

c

PTM	Sites	Peptides	Proteins
Phosphorylation	STY	527	279
Methylation	K	70	49
Dimethylation	K	29	22
Trimethylation	K	21	16
Acetylation	K	42	27
Formylation	K	53	28

Figure S2. Spectral library for single cell analysis of normal cholangiocytes and cholangiocarcinoma cell lines. (a) The number of proteins identified from DDA-PASEF runs of 20 cells, which resulted in a total of 4,584 proteins identified through protein database search using MSFragger. (**b)** Distribution of intensity-based absolute quantification (iBAQ) values of identified proteins. **(c)** Summary of PTMs identified from DDA-PASEF runs of 20 cells. Peptides containing C-terminal modified lysines were excluded from the search results.

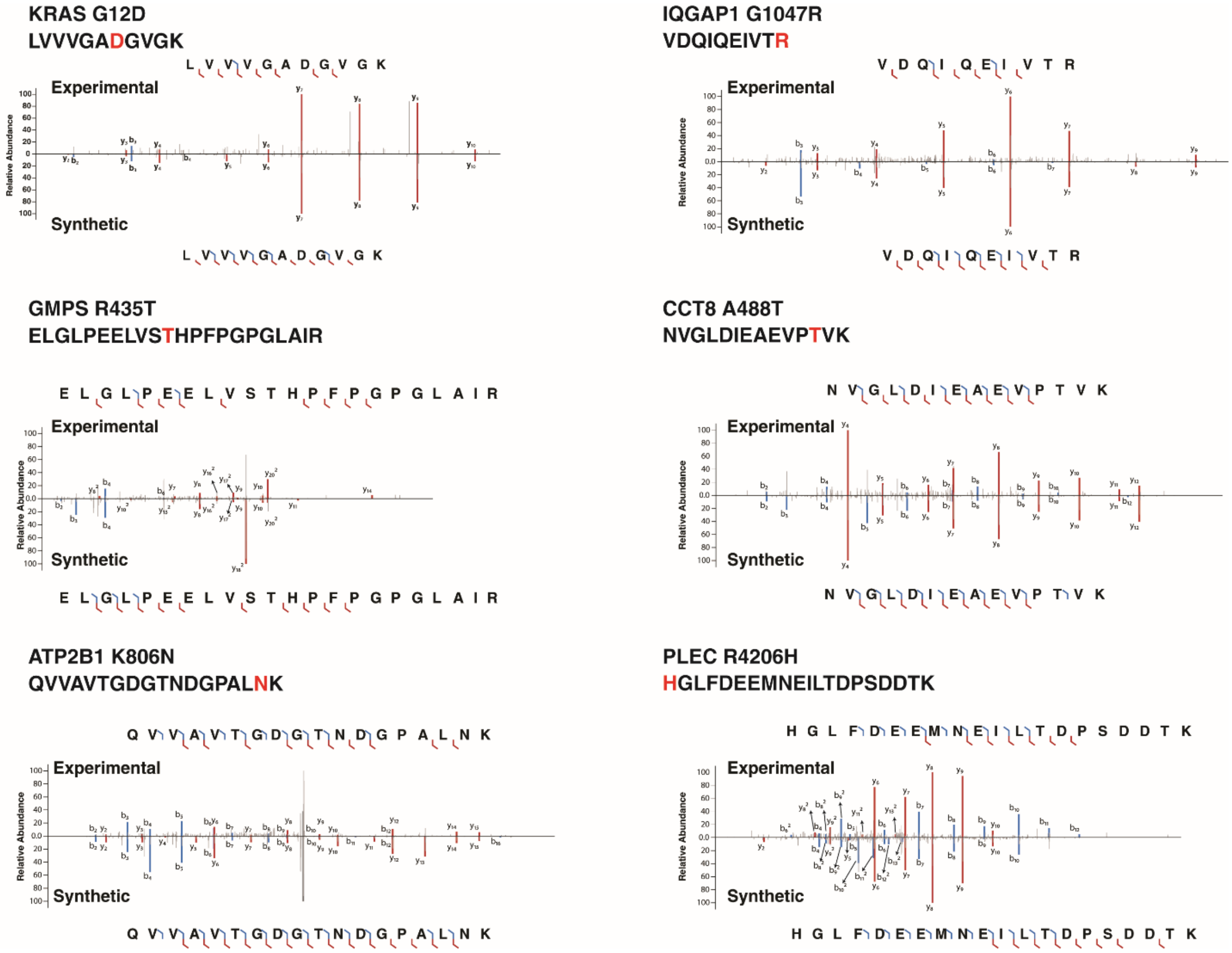

Figure S3. Annotated MS/MS spectra of peptides with SAPs. Identification of peptides with SAPs was confirmed using synthetic peptides. Synthetic peptides (1 fmol) were spiked into 1 ng of peptides digested from BSA and analyzed in DDA-PASEF mode. Amino acid highlighted in red indicates the SAP. The MS/MS spectra were plotted using a web-based tool Universal Spectrum Explorer (https://www.proteomicsdb.org/use/).

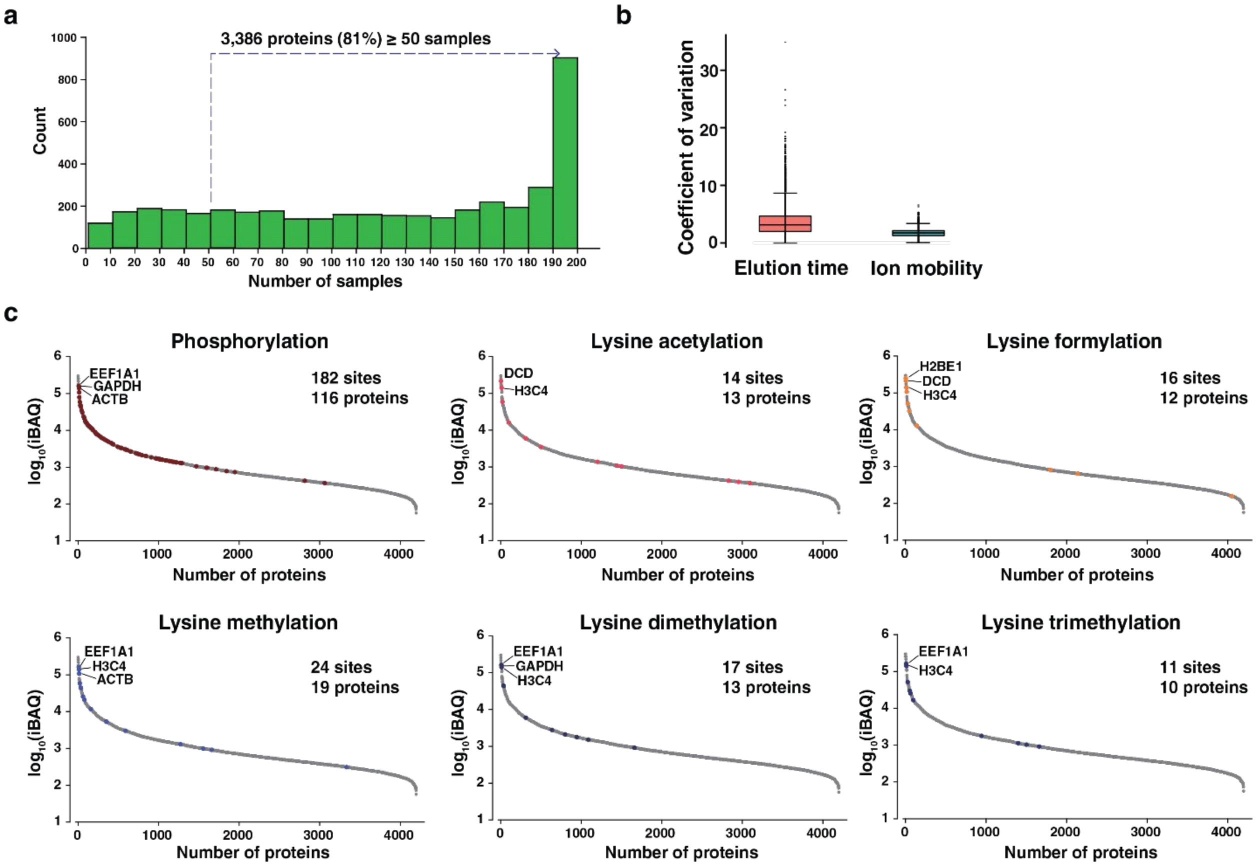

Figure S4. Analysis of individual single cells. **(a)** Data completeness showing distribution of proteins identified from multiple samples. Among 4,197 proteins, 3,386 proteins (81%) were detected in ≥50 single cell samples. **(b)** Distribution of coefficient of variation of elution time and ion mobility of identified peptides ≥2 samples (n=45,810). The boxplots show median, interquartile range (IQR), whiskers (1.5 x IQR) and outliers. **(c)** Waterfall plots of protein iBAQ intensities of 4,197 proteins with proteins containing PTMs marked in color. The number of modified sites and proteins is indicated.

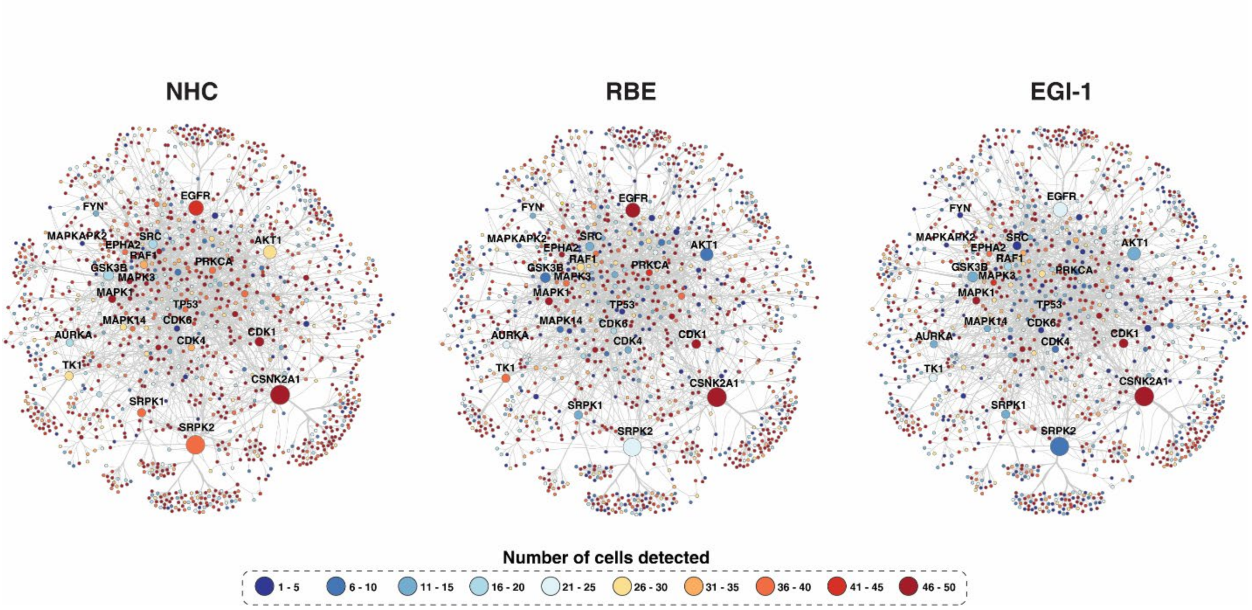

Figure S5. Single cell protein-protein interaction network. Kinase-centric protein-protein interaction network of NHC, RBE and EGI-1 cells. The node color indicates the number of cells in which the protein was detected.

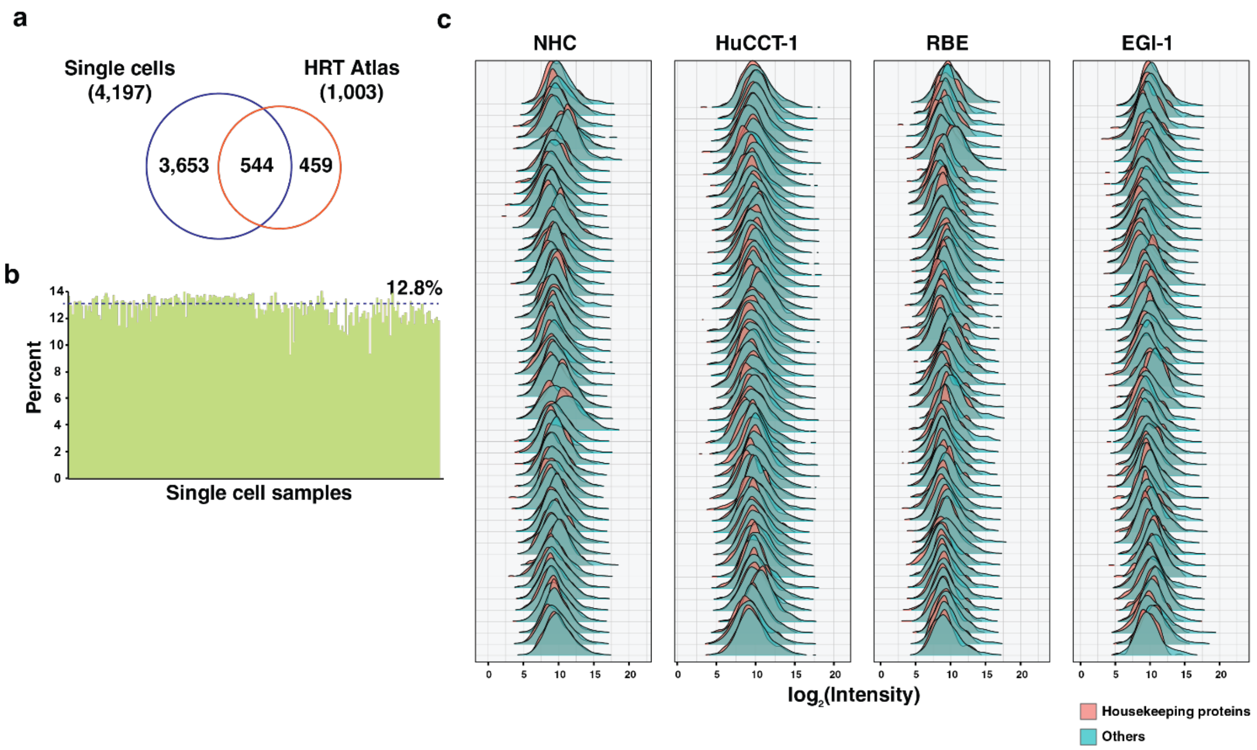

Figure S6. Housekeeping proteins identified from single cells. (a) Comparison of what with 1,003 housekeeping proteins curated at HRT Atlas resulted in 544 overlapping proteins. **(b)** Bar graph showing the percentage of housekeeping proteins in each of the single cell samples. Dashed line indicates average value. **(c)** Distribution of intensities of housekeeping proteins and the rest of proteins in each single cell.

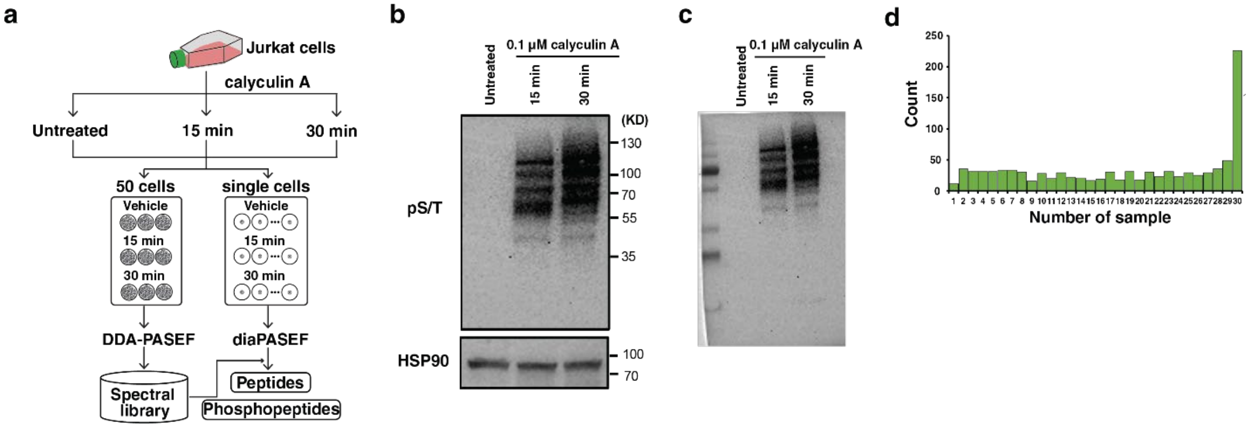

Figure S7. Analysis of phosphatase inhibitor treated single cells and tazemetostat-treated single nuclei. (a) Schematic workflow for single cell proteomics of calyculin A treated Jurkat cells. **(b)** Western blot of calyculin A treated conditions showing the increase in phosphorylation under calyculin A treatment. **(c)** Uncropped western blot image with size markers. **(d)** Data completeness showing distribution of proteins identified from multiple samples of single nuclei.
