# Supplementary material for: Diversity of post-translational modifications and cell signaling revealed by single cell and single organelle mass spectrometry
Source: Commun Biol. 2024 Jul 19;7:884. doi: 10.1038/s42003-024-06579-7 (PMC11271535; doi:10.1038/s42003-024-06579-7)
Supplement: Supplementary file 3 — Description of Supplementary Data [file 42003_2024_6579_MOESM3_ESM.pdf]

Description of Additional Supplementary Files

File name: Supplementary Data 1

Description: List of identified proteins from single cells of NHC, HuCCT-1, RBE and EGI-1 cell lines

File name: Supplementary Data 2

Description: List of peptides with SAPs along with their relative abundance

File name: Supplementary Data 3

Description: List of peptides with PTMs along with their relative abundance

File name: Supplementary Data 4

Description: List of peptides with PTMs along with their relative abundance

File name: Supplementary Data 5

Description: Numerical source data for graphs of main figures

File name: Supplementary Data 6

Description: Numerical source data for graphs of supplementary figures
